# Racial and ethnic differences in the risk of recurrent preterm or small for gestational age births in the United States: a systematic review and stratified analysis

**DOI:** 10.1186/s40748-024-00181-9

**Published:** 2024-06-03

**Authors:** Alka Dev, Justice Nagovich, Srinija Maganti, Elaina Vitale, Heather Blunt, Sophia E. Allen

**Affiliations:** 1 The Dartmouth Institute for Health Policy and Clinical Practice, Geisel School of Medicine, Dartmouth College, 1 Medical Center Drive, Lebanon, 03756 USA; 2 Biomedical Libraries, Dartmouth College, Hanover, NH USA; 3 Department of Obstetrics and Gynecology, Dartmouth Hitchcock Medical Center, Lebanon, USA

**Keywords:** Preterm birth, Low birthweight, Small for gestational age, Stillbirth, Neonatal mortality, Recurrence, Racial disparities

## Abstract

**Background:**

The risk of recurrent adverse birth outcomes has been reported worldwide, but there are limited estimates of these risks by social subgroups such as race and ethnicity in the United States. We assessed racial and ethnic disparities in the risk of recurrent adverse birth outcomes, including preterm birth, low birthweight, fetal growth restriction, small for gestational age, stillbirth, and neonatal mortality in the U.S.

**Methods:**

We searched MEDLINE, CINAHL Complete, Web of Science, and Scopus from the date of inception to April 5, 2022. We identified 3,540 articles for a title and abstract review, of which 80 were selected for full-text review. Studies were included if they focused on the recurrence of any of the six outcomes listed in the objectives. Study quality was assessed using the NIH Study Quality Assessment Tool. Heterogeneity across studies was too large for meta-analysis, but race and ethnicity-stratified estimates and tests for homogeneity results were reported.

**Results:**

Six studies on recurrent preterm birth and small for gestational age were included. Pooled comparisons showed a higher risk of recurrent preterm birth and small for gestational age for all women. Stratified race comparisons showed a higher but heterogeneous risk of recurrence of preterm birth across Black and White women. Relative risks of recurrent preterm birth ranged from 2.02 [1.94, 2.11] to 2.86 [2.40, 3.39] for Black women and from 3.23 [3.07, 3.39] to 3.92 [3.35, 4.59] for White women. The evidence was weak for race and ethnicity stratification for Hispanic and Asian women for both outcomes.

**Conclusions:**

Disparities exist in the recurrence of preterm birth, and race/ethnicity-concordant comparisons suggest race is an effect modifier for recurrent preterm birth for Black and White women. Due to the small number of studies, no conclusions could be made for small for gestational age or Hispanic and Asian groups. The results pose new research areas to better understand race-based differences in recurrent adverse birth outcomes.

**Supplementary Information:**

The online version contains supplementary material available at 10.1186/s40748-024-00181-9.

## Background

Women with an adverse birth outcome in an index pregnancy, including stillbirth, preterm birth (PTB), low birthweight (LBW), and neonatal mortality, are more likely to have a second adverse birth outcome than those with no previous adverse outcome [1, 2]. These findings indicate shared antecedents to adverse outcomes and the potential clustering of this outcome within mothers. Notable biological risk factors can be congenital, placental, and maternal and include preeclampsia, infection and inflammation, and vascular disease that can cause an adverse outcome in an index pregnancy but also persist or become exacerbated in between pregnancies to impact future births [3–5]. Recurrence risks have been documented among women with a prior adverse birth outcome in the United States (U.S.) but little is still known about the distribution of recurrence risk by race and ethnicity, even though adverse birth outcomes are consistently higher among Black women [6]. In the U.S., Black women are approximately 1.6 times more likely to have a PTB and two times more likely to have a stillbirth or neonatal death than their White counterparts [7–9]. Given the higher risk of adverse outcomes in an index pregnancy among Black women, we wanted to investigate whether the recurrence of these outcomes also varied by race and ethnicity. The aim of the review was to evaluate and summarize the findings of all relevant studies that presented race and ethnicity estimates for recurrence of any of our six selected outcomes: PTB, LBW, fetal growth restriction (FGR), small for gestational age (SGA), stillbirth, and neonatal mortality. Individual race or ethnicity in our study served as a proxy for potential racial and ethnic discrimination (interpersonal and structural) rather than as a direct cause of individuals’ birth outcomes [10].

## Methods

### Data sources

Two researchers (AD and JN) worked with two librarians (EV and HB) on conducting searches for relevant studies in MEDLINE (Ovid), CINAHL Complete (EBSCOhost), Web of Science (Clarivate Analytics), and Scopus (Elsevier) from the date of inception to April 5, 2022. A separate librarian reviewed all searches using a checklist modified from the Peer Review of Electronic Search Strategies (PRESS) [11]. To capture a wide range of studies, we utilized variations of keywords relating to PTB, LBW, SGA, FGR, stillbirth, neonatal mortality, and subsequent pregnancy. Subject headings and keywords were adapted to the syntax of each database. Studies were restricted to U.S. populations and English language only. We did not limit studies by year of publication or study design. Search results were downloaded to EndNote, and duplicate records were removed by HB and EV using EndNote’s automatic duplicate identification and then manually. The study was registered with PROSPERO (CRD42022320952). The complete search strategy is provided in Appendix A.

### Study selection

Studies were selected if they reported at least one of the six adverse birth outcomes of interest across two pregnancies. Adverse birth outcomes in this study were defined as PTB (delivery < 37 completed weeks of gestation); LBW (weighing < 2,500 g); SGA (< 10th percentile of babies of the same gestational age); FGR (fetal weight less than the 10th percentile for gestational age); stillbirth (fetal death occurring at 20 weeks of gestation or more); and neonatal death (deaths among live births during the first 28 completed days of life) unless specified otherwise. Other inclusion criteria included adult women (18 years of age or more for one of the pregnancies) and singleton pregnancies (for both the first and second pregnancies). We excluded studies that oversampled high-risk groups, tested an intervention, excluded Black people, did not disaggregate by race and ethnicity, or did not include a comparison group of healthy births.

Titles and abstracts were reviewed independently by teams of two reviewers using *Rayyan* to determine eligibility [12]. All reviewers were blinded to each other’s decisions. Each team independently screened one-half of the studies and determined their inclusion status (include, maybe, exclude). AD and JN worked as a team, and SM and SA worked as a team. Once completed, the review was unblinded, and each team met to resolve conflicts. For full-text review, the two teams switched their selections, i.e., each team reviewed studies short-listed by the other team. References of studies were evaluated to identify any that met the eligibility criteria. Decisions on studies considered for full-text review and any remaining disagreements were resolved through discussion among all four reviewers.

To assess the methodological quality of all included studies, our team used the NIH Study Quality Assessment Tool by the National Heart, Lung, and Blood Institute (NHLBI) and adapted it to focus on retrospective cohort studies [13]. Two independent reviewers rated each article; AD and SA worked as a team, and JN and SM worked as a team. This tool assessed the following components to judge methodological quality: exposure measures, outcome measures, study population, sample size, and statistical adjustments.

### Analysis

To facilitate a comparison of rates across studies, we calculated unadjusted risk ratios for studies that did not report pooled estimates per outcome definition. For example, Adams and colleagues reported adjusted odds ratios for early and late PTB. We combined raw data from their tables to produce a pooled unadjusted risk ratio for PTB less than 37 weeks [14]. Where reported, we report the published risk or odds ratios, whether adjusted or not. For each study, we also conducted race or ethnicity stratified analysis to investigate the association between an incident PTB or SGA and a subsequent PTB or SGA, respectively. We created a series of two-by-two tables showing the association and computed a weighted average of the risk ratios across the strata, as well as pooled Cochran-Mantel-Haenszel (CMH) estimates [15]. We calculated race or ethnicity stratified risk ratios and used the Woolf chi-square test to evaluate the homogeneity of stratified risk ratios. Data tables used to calculate estimates are included in Appendix 1.

We estimated pooled effect sizes by conducting a meta-analysis (using Mantel-Haenszel random effects modeling, unadjusted risk ratios (RR), and 95% CIs) for PTB recurrence for four articles, excluding a study from Missouri due to complete overlap in the study population with another. Since all studies were retrospective cohorts, we can assume that any observed variation was not due to the study design. We estimated pooled effect sizes for unadjusted estimates using the raw counts of births using RevMan 5.0 [16]. We reported the I^2^ statistic as a measure of heterogeneity across the studies and the funnel plot for potential bias [17].

## Results

### Description of studies

#### Study selection

**Fig. 1:**
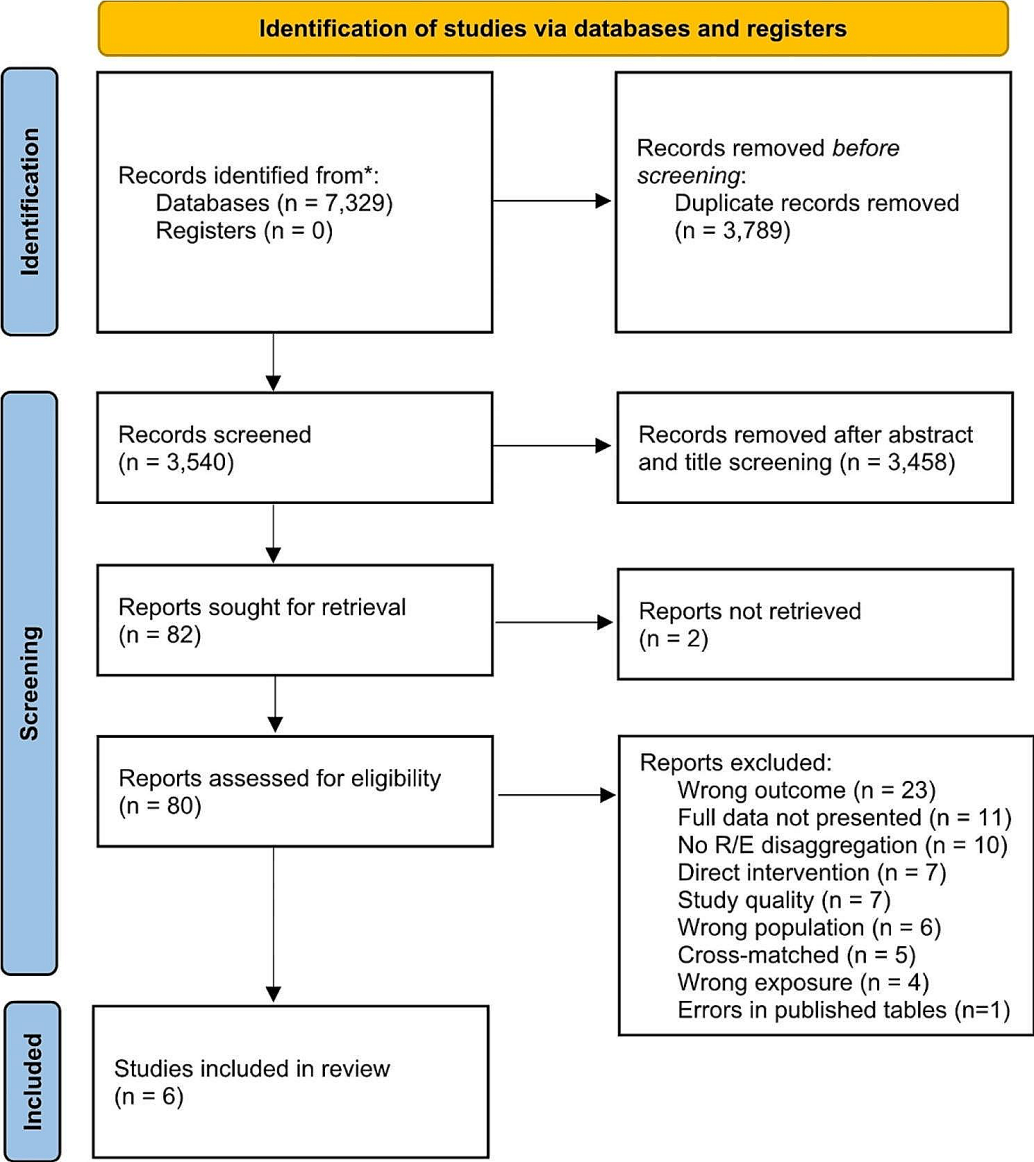
PRISMA 2020 flow diagram for new systematic reviews which included searches of databases and registers only^1^ ^1^ Page MJ, McKenzie JE, Bossuyt PM, Boutron I, Hoffmann TC, Mulrow CD, et al. The PRISMA 2020 statement: an updated guideline for reporting systematic reviews. BMJ 2021;372:n71. doi: 10.1136/bmj.n71

We identified 7,329 references through MEDLINE, CINAHL, Scopus, and Web of Science. After removing 3,789 replicates, 3,540 articles were examined for a title and abstract review. We did not find any studies that reported FGR. The title and abstract screen resulted in 82 studies for full-text review and quality assessment. Two references were conference abstracts; more information was requested from the authors but not received, leaving 80 studies for full-text consideration. In all, 74 studies were excluded after a full-text review due to a limited definition of the outcome (*n* = 23), lack of supporting data (*n* = 11), no disaggregation by race or ethnicity (*n* = 10), test of an intervention (*n* = 7), poor quality (*n* = 7), wrong population (*n* = 6), cross-matched outcomes (*n* = 5), wrong exposure (i.e., the outcome of the first birth was not one of our selected outcomes, *n* = 4), and significant errors in published data tables (*n* = 1), i.e. the sum of women for disaggregated racial and ethnic subgroups was higher (+ 13%) than the total number of women included in the study. Finally, six studies met our eligibility criteria and were included in our review (Fig. 1). For more information, refer to the Preferred Reporting Items for Systematic Reviews and Meta-Analyses (PRISMA) diagram [18]. 

#### Characteristics of included studies

All six were retrospective cohort studies published in peer-reviewed journals between 1998 and 2015 (Table 1). Three U.S. states were represented in the study population: Georgia, Missouri, and Utah. Additionally, one study evaluated more localized data in Chicago. Four of the six studies assessed the recurrence of PTB, and two looked specifically at the recurrence of SGA. Total births ranged from the smallest cohort, having only 1,522 births from Chicago to the largest cohort having 357,792 births from Georgia. While each study included mixed race and ethnicity populations and disaggregated data by White and Black race, two also disaggregated data by other races or Hispanic ethnicity, one each for PTB and SGA births. Additional characteristics can be found in Table 1.

**Table 1:** Characteristics of Included Studies

Author, year	State	Subjects (N)	Total Births	Race and Ethnicity (as designated in article)	Data Source	Study Period	Pooled Estimates [with 95% confidence intervals]
***Recurrent Preterm Birth (PTB)***							
Adams, 2000	Georgia	178,896	357,792	African American and White	Birth and death certificates	1980–1995	uRR = 2.85 [2.76, 2.94]
Ekwo, 1998	Chicago	761	1,522	African American and White	Hospital records from the Perinatal Network.	1988–1993	uRR = 2.92 [2.17, 3.93]
Grantz, 2015	Utah	25,820	51,640	Non-Hispanic White, Non-Hispanic Black, Hispanic, Asian/Pacific Islander, Other	NICHD Consecutive Pregnancies Study	2002–2010	uRR = 5.08 [4.64, 5.57]
McManemy, 2007	Missouri	19,025	38,050	19% Non-Hispanic Black 79% Non-Hispanic White 2% Other	Birth certificates	1989–1997	uRR = 2.09 [1.85, 2.36]
***Recurrent Small for Gestational Age (SGA)***							
Hinkle, 2014	Utah	25,241	50,482	Non-Hispanic White, Non-Hispanic Black, Hispanic, Asian/Pacific Islander, Other	NICHD Consecutive Pregnancies Study	2002–2010	uRR = 4.33 [3.92, 4.78]
Okah, 2010	Missouri	5,932	11,864	56% White 44% Black	Birth certificates	1995–2004	uRR = 3.91 [3.23, 4.59]

N = number; %=percent, PTB = Preterm Birth, SGA = Small for Gestational Age, uRR = unadjusted relative risk, aRR = adjusted relative risk, aOR = adjusted odds ratio

### Risk of bias of included studies

**Fig. 2:**
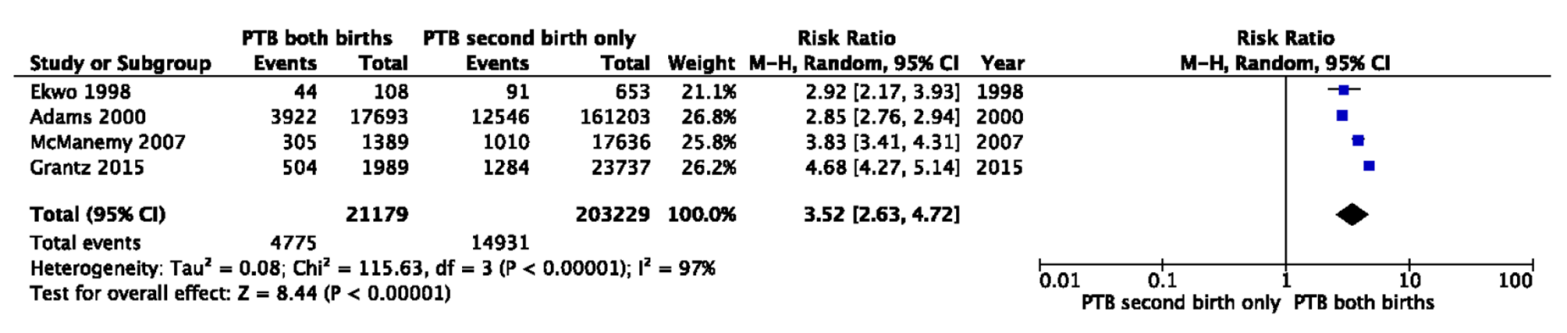
Risk of recurrent preterm birth

All six studies included multi-year birth cohorts from hospital and state-vital records or national studies led by the National Institute for Child Health and Development (NICHD). Therefore, the risk of bias due to participant inclusion criteria was minimal. Loss of follow-up did not introduce bias as all data were retrospective and only included people with at least two singleton births in the dataset. All studies had a quality score of seven or higher out of ten possible points. Despite substantive efforts to control for variation, heterogeneity remained consistently high (I^2^ = 97%; Fig. 2). We did not generate a funnel plot per recommendation, as less than 10 studies were included in the final review [17].

### Synthesis of results

#### Recurrent preterm birth

The most common combination of exposure and outcome in two consecutive pregnancies was PTB, covered in four studies (Table 1). Effect sizes for a recurrent PTB versus an incident PTB in the second pregnancy ranged from an unadjusted relative risk of 2.09 [1.85, 2.36] in the Missouri cohort covering the period 1989–1997 (*n* = 38,050 births) to an unadjusted relative risk of 5.08 [4.64, 5.57] in Utah state birth records covering the period 2002–2010 (*n* = 51,640 births) [19, 20]. All four PTB studies included Black and White populations, one of which also included Hispanic people and Asian/Pacific Islanders (Table 2). Our stratified estimates from all four studies show that the risk of a PTB in the second pregnancy was higher for both Black and White concordant groups when comparing those with a prior PTB to those without an index PTB. Ekwo and colleagues also presented adjusted odds ratios stratified by race which were similar (with overlapping confidence intervals to our unadjusted estimates [21]. The study reporting other subgroups showed a higher risk among Hispanic women (uRR = 3.83 [2.81, 5.21]) but not Asian-Pacific Islander women [20].

**Table 2:** Results of included studies by race and ethnicity

Author, year	Stratified race comparisons [95% confidence intervals]– calculated by study team	Race comparisons [95% confidence intervals]– as presented in paper
***Recurrent preterm birth (PTB)*** *Ref = no PTB in 1st pregnancy (unexposed*		
Adams, 2000	• Black: uRR = 2.02 [1.94, 2.11]* • White: uRR = 3.23 [3.07, 3.39]	• Stratified estimates for White and Black races presented for disaggregated maternal characteristics only (e.g. age groups)
Ekwo, 1998	• Black: uRR = 2.17 [1.56, 3.01]^NS^ • White: uRR = 3.65 [2.11, 6.32]	*Stratified estimates*: • Black: aOR = 2.98 [1.57, 5.66] • White: aOR = 4.26 [2.08, 8.70]
Grantz, 2015	• Asian - Pacific Islander: uRR = 1.81 [0.82, 4.04]* • Black: uRR = 5.77 [2.17, 15.4] ^NS^ • Hispanic: uRR = 3.83 [2.81, 5.21] ^NS^ • White: uRR = 4.87 [4.42, 5.38]	• Stratified estimates not presented. Comparisons to non-Hispanic White mothers showed no difference in adjusted relative risk for any race or Hispanic ethnicity.
McManemy, 2007	• Black: uRR = 2.86 [2.40, 3.39]* • White: uRR = 3.92 [3.35, 4.59]	• No estimates presented for race or ethnicity-based comparisons.
***Recurrent small for gestational age (SGA)*** *Ref = no-SGA in first pregnancy (unexposed)*		
Hinkle, 2014	• Asian uRR = 1.32 [1.26, 1.38]^NS^ • Black: uRR = 2.51 [1.08, 5.86]^NS^ • Hispanic uRR = 4.17 [3.14, 5.55]^NS^ • White: uRR = 4.44 [3.99, 4.95]	• Stratified estimates not presented. Comparisons to non-Hispanic White mothers showed no difference in adjusted relative risk for any race or Hispanic
Okah, 2010	• Black: uRR = 2.63 [2.22, 3.11]* • White: uRR = 5.36 [4.10, 6.99]	*Stratified estimates*: • Black: aRR = 2.66 [2.19, 3.23] • White: aRR = 5.37 [4.01, 7.18]

* *p* <.05 for Woolf test– stratification by race/ethnicity recommended, NS = not significant for Woolf test– can pool race subgroups,, uRR = unadjusted relative risk, aRR = adjusted relative risk, aOR = adjusted odds ratio

In two studies of recurrent PTB, the Woolf test for heterogeneity was significant for stratified Black and White risk ratios, showing that unadjusted Black and White risk ratios were not equal (Table 2). In these studies, the stratified unadjusted risk ratios were 2.02 [1.94, 2.11] and 2.86 [2.40, 3.39] for the risk of a recurrent PTB among Black women and 3.23 [3.07, 3.39] and 3.92 [3.35, 4.59] among White women. However, in the other two studies by Grantz et al. and Ekwo et al., the stratified risk ratios were not significantly different between Black and White mothers. Thus, the role of race in modifying the risk from an incident PTB to a subsequent PTB was unclear. In the study by Grantz and colleagues, the stratified risk ratios for Black and White women were equivalent, i.e., the Woolf test for homogeneity was not significant, but the study had a very small sample of Black births and wide confidence intervals [20]. Grantz also studied recurrent PTB among Hispanic and Asian women, finding that the stratified risk ratio for Asian women was not significant, i.e., Asian women did not have a higher risk of a second PTB when comparing those with a prior PTB to those with a previous term birth. However, the stratified risk ratio for Hispanic women was significant (uRR = 3.83 [2.81, 5.21], *p* <.001) but not statistically different from the estimate for White women, i.e. Hispanic ethnicity did not modify the relationship between the exposure (prior PTB) and the outcome (subsequent PTB). Risk ratios for recurrent PTB were statistically different for both Hispanic and Asian women compared to White women in the only other study to look at these differences.

#### Recurrent SGA

Two studies investigated recurrent SGA (Table 1). The risk of recurrence of an SGA birth was 4-fold higher than the risk of an incident SGA in the second pregnancy in Georgia vital records of 50,482 births from 1980 to 1995, and Missouri vital records of 11,864 births from 1995 to 2004 [22, 23]. Both studies disaggregated results by race and ethnicity for SGA (Table 2). The NICHD data showed significant differences by race and ethnicity for recurrent SGA for all races and Hispanic ethnicity [22]. The Missouri cohort showed that both Black and White mothers had a higher risk of a recurrent SGA, ranging from a relative risk of 2.63 [2.22, 3.11] for Black mothers in Missouri to a relative risk of 2.53 [1.08, 5.91] among Black mothers in the NICHD cohort. For White mothers, these risks were 5.35 [4.10, 6.91] and 4.44 [3.99, 4.94], respectively, in Missouri and the NICHD cohort.

The Woolf test for homogeneity of stratified risk ratios from Hinkle et al. showed that they were not statistically different for any race or ethnicity, and therefore, data could be pooled to estimate the risk of recurrent SGA [22]. However, the test for homogeneity in Okah et al. showed that the stratified estimates for Black and White women were statistically different [23]. They did not look at other races or Hispanic ethnicity. Okah and colleagues also presented adjusted risk ratios for recurrent SGA stratified by race which were similar, with overlapping confidence intervals to our unadjusted estimates [23].

## Discussion

In line with existing reviews and international studies, we found a higher risk of recurrence of PTB and SGA overall. We found that effect sizes for recurrent PTB generally ranged from two to five times higher than incident PTB. Similarly, a four-fold risk was observed for recurrent SGA in two studies. We did not find any U.S. studies on the recurrence of any other outcomes. With respect to race and ethnicity comparisons, the results were not consistent in showing a higher risk of recurrence across Black race and Hispanic ethnicity, and they were inconclusive for Asian and Pacific Islander mothers. Using the Woolf test for homogeneity of race and ethnicity-stratified risk ratios, we found evidence of effect modification by race for the risk of a recurrent PTB among Black and White mothers in two of four studies only, and the evidence regarding race as a modifier was weak for SGA in the same. While comparisons of Black and White births suggested a higher recurrent PTB risk among Black mothers, race-stratified results also indicated that recurrent PTB could be an equally severe issue for White mothers. Stratified estimates for three of the four studies showed that Black mothers’ risk of a recurrent PTB was relatively lower than that of White mothers, i.e., Black mothers with a prior PTB had a smaller risk ratio of a second PTB than White mothers, pointing to the potential effect of higher risk in the first PTB, which, in turn, affects their likelihood of experiencing another preterm birth. One reason for observing a lower recurrence risk among Black women might also be fetal loss due to their heightened risk for adverse birth outcomes [24].

Only one study produced estimates for Hispanic and Asian mothers, finding that Hispanic women experience a higher risk if they had a prior PTB compared to a prior term birth. However, this risk is similar to that of White mothers. For Asian women, the risk was not higher for a subsequent PTB given a prior PTB. The stratified effect sizes varied across the groups and produced inconsistent evidence of Black race as a factor in recurrent PTB. For SGA, the stratified analysis showed that risk was higher for all races and Hispanic ethnicity, but one study found these risks to be similar to those of White women, i.e., pooled estimates would be recommended, but another showed statistically different estimates for Black and White women. Without data for covariates, these comparisons were largely unadjusted. None of the international studies made any comparisons by socioeconomic status or other socially salient categories such as race and ethnicity in the U.S. Data of PTB by race and ethnicity in the U.S. has established clear evidence of higher risk among Black women but evidence regarding higher risk among Asian or Hispanic women is less consistent [24]. Our research similarly found that Asian women’s risk of either PTB or SGA was not different from that of White women, but for Hispanic women, the stratified risk ratios were statistically different from White women.

Research also suggests a genetic link between repeat adverse outcomes, especially across more than two pregnancies, given the likelihood of the same mother delivering a preterm baby at the same gestation over consecutive pregnancies [34]. The authors argue that the likelihood of similar social and environmental exposures occurring at the same time in consecutive pregnancies is slim, suggesting an underlying genetic pathology. Similarly, genetic risk may differentially predispose Black and White women to heightened inflammatory response to infection during pregnancy [26]. However, to our knowledge, no study has provided a direct genetic analysis of recurrent preterm birth, even in the absence of other risk factors. Furthermore, given the legacy of anti-Black racism in the U.S., and lower rates of preterm birth among foreign-born Black women, relying on genetic risk to explain the Black-White disparity in the recurrence of adverse birth outcomes is not recommended [27].

Our findings were limited in the range of outcomes from similar studies using national registries and hospital data from Europe (Sweden, Finland, Malta, Scotland, Norway) [28–31], Australia [32], India [33], Indonesia [34], Brazil [35], Tanzania [36], and several other developing countries [37], which have shown that having a stillbirth, PTB, LBW, or neonatal mortality increases the risk of having another similar outcome. Further, we excluded recurrent cross-matched outcomes due to poor reporting of race and ethnicity measures. However, other published research has also shown higher risks for adverse cross-outcomes across pregnancies. A 2017 review and meta-analysis by Malcova and colleagues (17 studies) found an increased risk of stillbirth, PTB, and SGA if a previous birth had resulted in any of the outcomes, with higher risk by the degree of severity of the prior outcome [2]. Meta-analyses should only be conducted if the statistical combination of individual study results is meaningful. Metelli and Chaimani (2020) describe the challenges of running a meta-analysis with observational study data, including increased risk of reporting and publication bias, high heterogeneity, and confounding [38]. Our study reported too a high level of heterogeneity to draw conclusions from the meta analysis.

We extend some caution that the studies in the U.S. are based on a limited number of states, have overlapping cohorts, and vary significantly in study design and population coverage. For example, the two studies disaggregating data for Hispanic and Asian mothers had a relatively small sample of Black women (∼ 100 births each) from the NICHD cohort for the same period. Moreover, all comparisons of effect sizes should be made with caution due to the variation in covariates, sample size for underlying risk adjustments, and health system capacity across studies. Any conclusions about the risk of recurrence and repeat PTB and SGA events in the U.S. should be avoided unless estimates can be made across adequately powered samples of racially and ethnically diverse populations and stratified analysis is produced.

A strength of our study is that it is the only review in the U.S. or internationally that reports findings for socially salient categories such as race and ethnicity, which begins to consider the relevance and importance of maternal health disparities across these groups. For example, while we found evidence of recurrence risk for PTB across all races and Hispanic ethnicity, these differences were also conditional on the risk of an incident PTB, which is known to be higher among Black women in the U.S. compared to all other racial or ethnic categories. Therefore, recurrence risk is not indicative of the overall risk for an adverse outcome such as PTB. Similar disparities will likely exist in other countries, whether by race, ethnicity, or socioeconomic status, which is important for global maternal health equity. Second, the outcome of each pregnancy in these studies was established through direct measurement, i.e., from a record of each birth itself through either vital or hospital records. As Adams 2001 found, relying on data from the second pregnancy’s birth certificate for the outcome of the first pregnancy will grossly undercount adverse outcomes in the first pregnancy, and direct measurement at the time of birth is recommended [39].

A limitation of this systematic review was high heterogeneity influenced by factors such as differences in underlying study populations, study inclusion and exclusion criteria, adjusted variables, and analytical methods. For this reason, we chose not to present the results of our meta-analysis. Possible reasons for high heterogeneity could be the wide variation in sample sizes and underlying populations (especially by race and ethnicity), adjustments for underlying medical conditions and comorbidities, setting, period and duration of data collection, and varying exposure and outcome definitions (e.g., spontaneous versus indicated PTB). This heterogeneity can be addressed with more information on the characteristics of the included populations in each study. Overall, we questioned the validity of conducting a meta-analysis given persistent heterogeneity despite attempts to adjust for study differences and confounding variables. This limitation reflects the quality of included studies and the nature of combining observational studies versus RCTs rather than an inherent flaw in our methods. Our conclusions are also limited by the lack of adjusted estimates that fit our research question. While the chosen studies aligned with our selection criteria, they provided results that limited comparisons by race and ethnicity. For example, we decided to produce stratified unadjusted estimates from study tables to make comparisons across the studies since these were either not provided or were presented with varying levels of disaggregation for maternal age, type of PTB (early or late), and periods. As noted earlier, we were disappointed to have to drop one study with a large sample size due to unresolvable errors in the published table.

## Conclusions

Race, and especially race-based social inequities, continue to be a persistent factor in inequities experienced by Black women in the U.S. However, the influence of race in the recurrence of adverse outcomes needs a better evidence base. Future research should focus on the clinical and psychosocial risk factors that could predict recurrent adverse birth outcomes in the U.S. Research in this area can provide more insight into the shared causal mechanisms that increase the risk of recurrent adverse birth outcomes for everyone. Further, studies using racially and ethnically diverse datasets with robust sample sizes should be conducted to understand the disparities across these groups better. While the number and quality of these studies is insufficient to conclude whether recurrence will occur along the same lines of racial inequity as incident cases, the findings reported here suggest sufficient complexity in their results to warrant a comprehensive examination of recurrence in nationally representative datasets. In the absence of a national birth registry or other longitudinal birth data sources, it would be nearly impossible to make these calculations. This is a significant gap considering the size of our healthcare system and the scale of racial inequities in maternal health outcomes. Maternally linked patient and birth records must be made more accessible across more states to generate datasets that will facilitate this research.

### Electronic supplementary material

Below is the link to the electronic supplementary material.

Supplementary Material 1

## Data Availability

All data tables used to estimate effects are provided in Appendix 1.

## Abbreviations

CMH Cochran: Mantel-Haenszel
FGR: Fetal Growth Restriction
LBW: Low birthweight
NHLBI: National Heart, Lunch, and Blood Institute (NHLBI)
OR: Odds Ratio
PRESS: Peer Review of Electronic Search Strategies
PTB: Preterm birth
RR: Risk Ratio or Relative Risk
SGA: Small for gestational age
